# PHD and TFIIS-Like Domains of the Bye1 Transcription Factor Determine Its Multivalent Genomic Distribution

**DOI:** 10.1371/journal.pone.0102464

**Published:** 2014-07-16

**Authors:** Marina Pinskaya, Yad Ghavi-Helm, Sylvie Mariotte-Labarre, Antonin Morillon, Julie Soutourina, Michel Werner

**Affiliations:** 1 iBiTec-S CEA, FRE3377, Gif-sur-Yvette, France; 2 CNRS, FRE3377, Gif-sur-Yvette, France; 3 Université Paris-Sud, FRE3377, Gif-sur-Yvette, France; 4 ncRNA, epigenetic and genome fluidity, Institut Curie, Centre de Recherche, CNRS UMR3244, Université Pierre et Marie Curie, Paris, France; Southern Illinois University School of Medicine, United States of America

## Abstract

The BYpass of Ess1 (Bye1) protein is a putative *S. cerevisiae* transcription factor homologous to the human cancer-associated PHF3/DIDO family of proteins. Bye1 contains a Plant Homeodomain (PHD) and a TFIIS-like domain. The Bye1 PHD finger interacts with tri-methylated lysine 4 of histone H3 (H3K4me3) while the TFIIS-like domain binds to RNA polymerase (Pol) II. Here, we investigated the contribution of these structural features to Bye1 recruitment to chromatin as well as its function in transcriptional regulation. Genome-wide analysis of Bye1 distribution revealed at least two distinct modes of association with actively transcribed genes: within the core of Pol II- and Pol III-transcribed genes concomitant with the presence of the TFIIS transcription factor and, additionally, with promoters of a subset of Pol II-transcribed genes. Specific loss of H3K4me3 abolishes Bye1 association to gene promoters, but doesn’t affect its binding within gene bodies. Genetic interactions suggested an essential role of Bye1 in cell fitness under stress conditions compensating the absence of TFIIS. Furthermore, *BYE1* deletion resulted in the attenuation of *GAL* genes expression upon galactose-mediated induction indicating its positive role in transcription regulation. Together, these findings point to a bimodal role of Bye1 in regulation of Pol II transcription. It is recruited *via* its PHD domain to H3K4 tri-methylated promoters at early steps of transcription. Once Pol II is engaged into elongation, Bye1 binds directly to the transcriptional machinery, modulating its progression along the gene.

## Introduction

In eukaryotes, transcription is finely regulated through complex mechanisms, which promote RNA polymerases recruitment and progression through a chromatin matrix leading to efficient RNA synthesis. *In vitro* and *in vivo* studies indicate that Pol II movement is discontinuous and that nucleosomes provoke its pausing and backtracking during transcription at promoter-proximal regions and within the body of genes [1]–[3]. After backtracking, arrested Pol II is reactivated by the elongation factor TFIIS that binds through its domain II and RSADE structural modules to the jaw and funnel domains of Pol II, thereby enhancing the enzyme’s intrinsic RNA cleavage activity [4]. Escape from backtracking is essential for efficient transcript elongation and, in general, for cell viability [1], [5], [6]. In addition, several studies revealed that TFIIS is associated with gene promoters and acts during preinitiation complex assembly [7]–[10]. TFIIS has also been shown to participate in Pol III transcription. It is associated with the Pol III machinery at the majority of class III genes in yeast and mouse embryonic stem cells [11], [12]. Altogether, these findings indicate that TFIIS acts as a positive transcription factor stimulating both Pol II and Pol III transcription and, hence, is implicated in many physiological processes.

In *S. cerevisiae,* the Pol II-interacting domain II of TFIIS shares a high homology with a central TFIIS-like domain (aa 232–365) of the putative transcription factor Bye1 that was discovered as a multicopy suppressor of the *ESS1* gene deletion. *ESS1* encodes a highly conserved peptidyl-prolyl isomerase implicated among other functions in the regulation of the whole transcriptional process, stimulating serine 5 dephosphorylation of the Pol II C-terminal domain repeats during transcription elongation [13]–[15]. According to genetic studies, Ess1 opposes positive effects of TFIIS and of the Spt4-5 complex, promoting transcriptional pausing or arrest [15], [16]. Bye1, when expressed at high levels from a multicopy plasmid, is able to suppress Ess1 dysfunction but renders cells more sensitive to a transcription elongation inhibitor, 6-azauracil [15]. These findings suggest that Bye1 might modulate Pol II progression through gene bodies. Recent crystallographic studies have shown that the TFIIS-like domain of Bye1, as that of TFIIS, is able to bind directly to the jaw domain of Pol II [17]. In addition, Bye1 contains a PHD finger (aa 74–134) and a SPOC domain (aa 447–547). SPOC domains have been implicated in protein-protein interactions [18]. The PHD finger of Bye1 has been shown to bind specifically to H3K4me3 *in vitro* and, globally, *in vivo* nearby the transcriptional start site (TSS) of actively transcribed loci [17]. However, a role of Bye1 binding to Pol II and modified histone H3 and its consequences on transcription remain elusive.

To better understand Bye1 function in transcription and its relation to Pol II and chromatin modifications *in vivo*, we applied molecular and genetic approaches. Genome-wide analysis of Bye1 localization revealed that Bye1 is associated with bodies of Pol II and Pol III-transcribed genes (class II and III genes, respectively) with distribution profiles resembling that of TFIIS. In addition, Bye1 is present at promoters of a subset of actively transcribed class II genes. This location depends on the presence of the H3K4me3 mark. We confirmed *in vivo* that Bye1 physically interacts with the jaw domain of Pol II and necessitates PHD, TFIIS-like and SPOC domains for strong binding. In general, *BYE1* deletion alone has no consequence on cell viability. However, upon osmotic stress or carbon source changes, it becomes indispensable for normal growth and efficient transcriptional activation. We propose that Bye1 uses two distinct modes of recruitment to chromatin: through the binding to H3K4 tri-methylated nucleosomes and/or to the Pol II machinery. Hence, it is involved in regulation of transcription in at least two steps: (i) very early in the transcriptional cycle when it is recruited to the H3K4me3 mark around TSS and/or (ii) during elongation through interaction with Pol II in the body of genes. The two modes of Bye1 action might occur at the same genomic loci and have a positive outcome on the transcriptional process stimulating Pol II progression through the gene.

## Results

### Bye1 is associated with Pol II- and Pol III-transcribed genes *in vivo*

**Figure 1:**
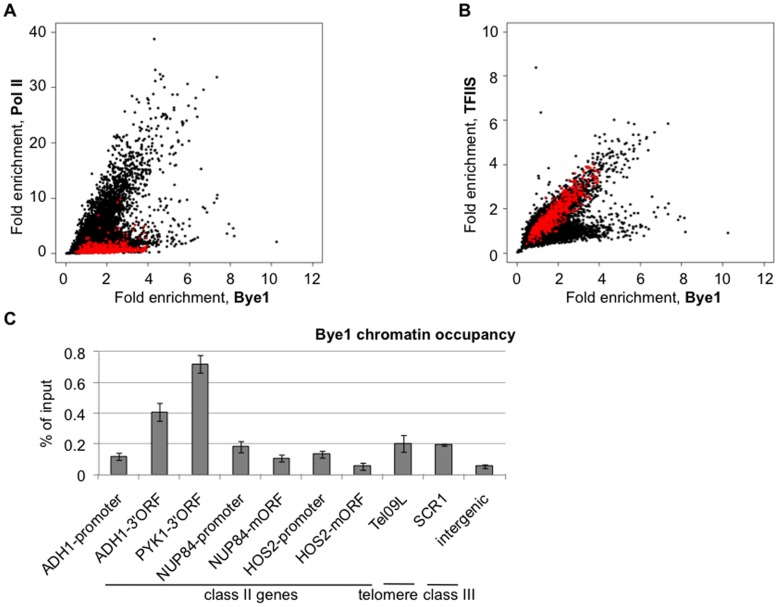
Analysis of Bye1, TFIIS and Pol II chromatin occupancies in yeast: (A, B) Genome-wide scatter-plots of Bye1 and Pol II distribution assessed by ChIP-chip in the Bye1-Myc HA-TFIIS strain. Comparison of fold enrichment (**A**) between Pol II and Bye1 and (**B**) between TFIIS and Bye1. Red dots correspond to probes complementary to class III genes (listed in Table S7); (**C**) Quantification of Bye1 enrichment in coding regions of class II and class III genes, promoters of some class II genes and Y’-containing telomeric ends by ChIP. 3′ORF and mORF stand for the 3′end and middle coding regions.

To better understand a role of Bye1 in transcription, we performed chromatin immunoprecipitation assays followed by hybridization to microarrays (ChIP-chip) assessing in parallel genome-wide distributions of the HA-tagged TFIIS, the Myc-tagged Bye1 and Pol II in *S. cerevisiae*. All oligonucleotides significantly enriched in Bye1-bound chromatin (fold enrichment ≥2, p value ≤0.05) were further divided into three separate groups: (1) 4075 probes corresponded to coding regions of Pol II-transcribed genes with occupancy by all three proteins: Pol II, Bye1 and TFIIS; (2) 130 oligonucleotides enriched in TFIIS and Bye1 bound chromatin but not in Pol II; and, finally, (3) 387 oligonucleotides hybridized uniquely to Bye1 bound chromatin (Figure 1, Table S1–S3, Figure S1–S2).

The first group represents actively transcribed class II genes, since all these loci showed high presence of Pol II (Figure 1A, Figure S1). The Pol II occupancy was concurrent with those of TFIIS and Bye1. The second group of Bye1 bound loci was enriched in TFIIS, but not in Pol II (red dots, Figure 1A). The analysis revealed that the majority of these probes corresponded to class III genes transcribed by Pol III (Figure S2). To confirm this finding, we compared Bye1 distribution with that of TFIIS. Both proteins showed association with class II and class III genes (black and red dots, respectively) (Figure 1B). In addition, another subset of loci showing co-localization of Bye1 and TFIIS was deprived of Pol II and corresponded to the very extremities of either X or Y′ telomeric elements, just upstream of telomeric repeats (Table S1–S3). The significance of Bye1 and TFIIS association with telomeres was not investigated further. Finally, the last group of probes exclusively enriched by Bye1 corresponded to promoters and early 5′ coding regions of a subset of Pol II-transcribed genes (536 genes, in total) (Table S4, Figure S1). Importantly, all of these genes are transcribed in the given experimental conditions, though at a low level, since PoI II is hardly detectable within gene bodies (Table S1, S3). According to a Gene ontology analysis, this subset includes genes coding for proteins involved in RNA metabolism, transport and transcription (Table S5).

The results obtained by ChIP-chip experiments were confirmed by conventional ChIP on a number of Pol II- and Pol III-transcribed genes (Figure 1C). All selected genes indeed displayed a significant Bye1 occupancy within their coding regions and a subset of protein-coding genes showed low but measurable Bye1 enrichment at promoters when compared to an intergenic non-transcribed region used as a negative control. Together these data indicated the association of Bye1 with chromatin in at least two distinct contexts: promoters of a subset of class II genes and bodies of all transcribed class II and class III genes.

### The presence of Bye1 at promoters and coding regions of class II genes requires active transcription

**Figure 2:**
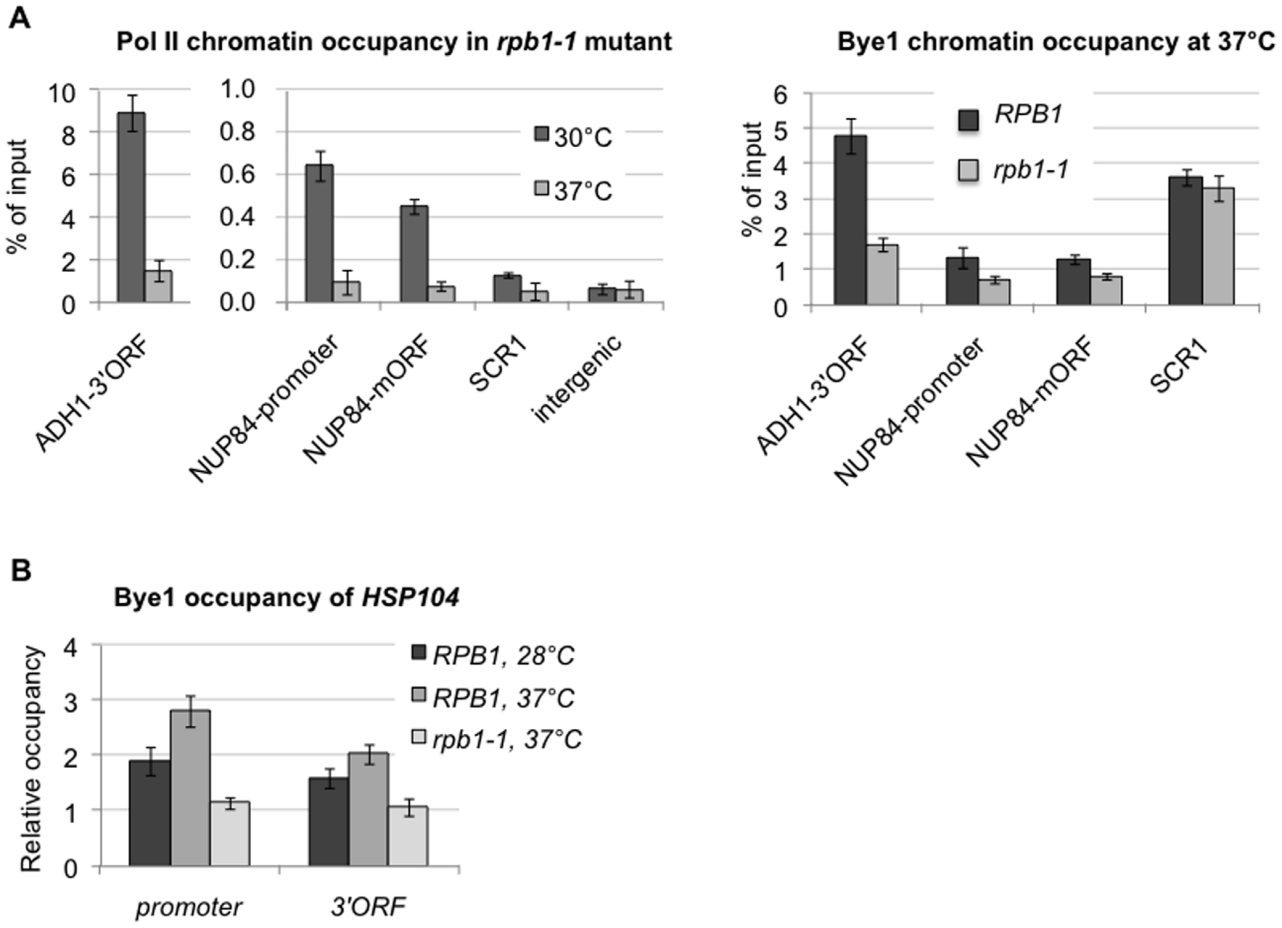
Bye1 association with class II genes requires active transcription: (A) Quantification of Pol II enrichment in *rpb1-1* mutant and Bye1-Myc enrichment in *RPB1 WT* and *rpb1-1* strains at 30°C and 37°C by ChIP; (B) Quantification of Bye1 enrichment at the heat-induced *HSP104* locus at 28°C and 37°C by ChIP. An intergenic genomic locus is used for a relative occupancy calculation; 3′ORF and mORF stand for the 3′end and middle coding regions.

To investigate a dependence of Bye1 recruitment to chromatin on active Pol II transcription, we followed Bye1 occupancy at promoters and coding regions of class II genes by ChIP in a strain expressing the *rpb1-1* Pol II thermosensitive allele transformed either with an empty vector or a vector expressing the wild-type Rpb1 protein. We found that shifting the strain expressing *rpb1-1* to 37°C for 30 min caused a dissociation of Pol II from coding regions of genes pointing to abolishment of transcription and, as a consequence, it resulted in a loss of Bye1 recruitment to both promoters and coding regions of class II genes (Figure 2A). Importantly, total amount of the Bye1 protein and Bye1 occupancy of the *SCR1* class III gene remained unchanged under the same conditions (Figure 2A and S3). This experiment indicates that Bye1 is not associated with class II genes when their transcription is interrupted. The heat shock gene *HSP104* is transcribed at a low basal level at permissive temperature (28°C) but is very quickly induced upon heat shock (37°C). Using ChIP, we showed that Bye1 is present at the *HSP104* promoter and within the gene at 28°C and 37°C but its occupancy was increased at the high temperature suggesting that the enrichment of Bye1 as that of Pol II correlates with the transcription level (Figure 2B). We separated class II genes in three categories: with high, middle and low levels of expression corresponding to more than 10, from 1 to 10 and less than one transcripts per cell, respectively [19]. Correlation analysis of Bye1 and Pol II enrichments for these three categories of genes confirmed that Bye1 recruitment to chromatin is transcription-dependent (Figure S4). Indeed, we showed that Bye1 mean fold enrichment was higher and Pearson (*r*) and Spearman (*ρ*) correlation coefficients between Bye1 and Pol II were improved with the increase of gene expression: (1) *r* = 0.545, *ρ* = 0.547 for lowly expressed, (2) *r* = 0.605, *ρ* = 0.635 for mildly expressed, and (3) *r* = 0.861, *ρ* = 0.846 for highly expressed genes (Figure S4). For lowly and mildly expressed genes, the worst correlated cases corresponded in majority to early 5′ coding regions of class II genes also showing Bye1 at promoters. Notably, we observed the same phenomenon for TFIIS and the strongest correlation between TFIIS and Bye1 within class II genes was observed for the highly expressed genes (*r* = 0.905, *ρ* = 0.867).

Hence, Bye1 recruitment to chromatin is dependent on active Pol II transcription and strongly correlates with Pol II presence and gene transcription within highly expressed genes.

### Bye1 interacts with Pol II *in vivo*

**Figure 3:**
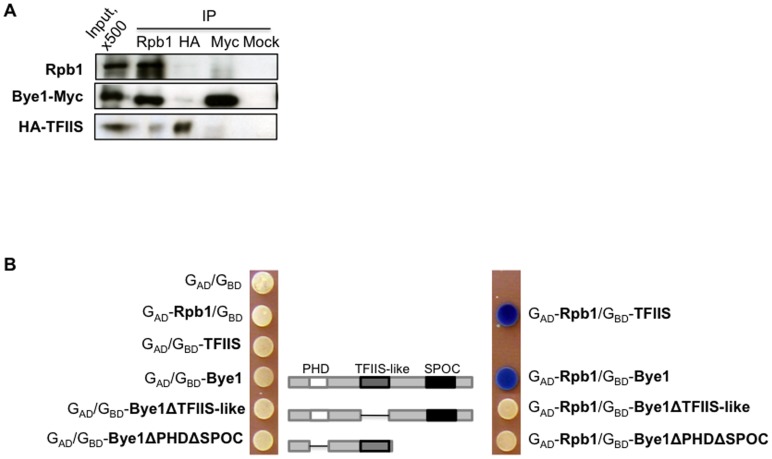
Bye1 is associated with the Pol II Rpb1 subunit *in vivo*: (A) Co-immunoprecipitation of Bye1-Myc, HA-TFIIS and Rpb1 from cross-linked cellular extracts; (B) Bye1 and TFIIS interact with the jaw domain (aa 1154–1252) of Rpb1 by Y2H. All three domains, TFIIS-like, PHD and SPOC, are essential for Rpb1-Bye1 interaction: overlay of the Y190 strain transformed with either empty G_AD_/G_BD_ vectors or G_AD_ fused to the Rpb1 jaw domain and G_BD_ fused to Bye1 or TFIIS. Left panel: control for the autoactivated β-galactosidase activity. Right panel: interactions are observed for Rpb1 and TFIIS and for Rpb1 and Bye1 the whole and the PHD and SPOC deleted proteins.

The Bye1 TFIIS-like domain (aa 232–354) presents high homology with the domain II of TFIIS (aa 146–247). The observed co-localization of Pol II, TFIIS and Bye1 within a large number of transcribed regions suggested that, similarly to TFIIS, Bye1 may bind to the transcriptional machinery *in vivo*. We investigated this hypothesis by co-immunoprecipitation (IP) of Pol II and Bye1 from formaldehyde cross-linked chromatin (Figure 3A). Immunoprecipitated Rpb1 retained Bye1 and, at a low level, TFIIS, suggesting association of the two proteins with Pol II (Figure 3A). On the contrary, immunoprecipitated Bye1 or TFIIS did not show detectable Pol II, possibly because of IP and Western blot technical limits. To further assess the ability of Bye1 to bind to Rpb1 *in vivo,* we performed a yeast two-hybrid assay (Y2H). Y190 strains transformed by empty vectors or vectors encoding fusions of the Gal4 activation domain (G_AD_) with the jaw domain of Rpb1 and fusions of the Gal4 DNA-binding domain (G_BD_) with Bye1 or TFIIS were overlaid with X-Gal agarose to assess β-galactosidase activity. As expected, both TFIIS and Bye1 showed interaction with the jaw domain of Rpb1 *in vivo* (right panel, Figure 3B). Then, we performed the same assay with Bye1 lacking PHD and SPOC (Bye1ΔPHDΔSPOC) or the TFIIS-like domain alone (Bye1ΔTFIIS-like). Interestingly, the TFIIS-like domain is essential, but not sufficient for interaction with the Rpb1 jaw suggesting that the PHD and/or SPOC domains somehow contribute to strong Pol II binding *in vivo* (Figure 3B).

### Binding of Bye1 to class II genes promoters requires Set1C-mediated tri-methylation of H3K4

**Figure 4:**
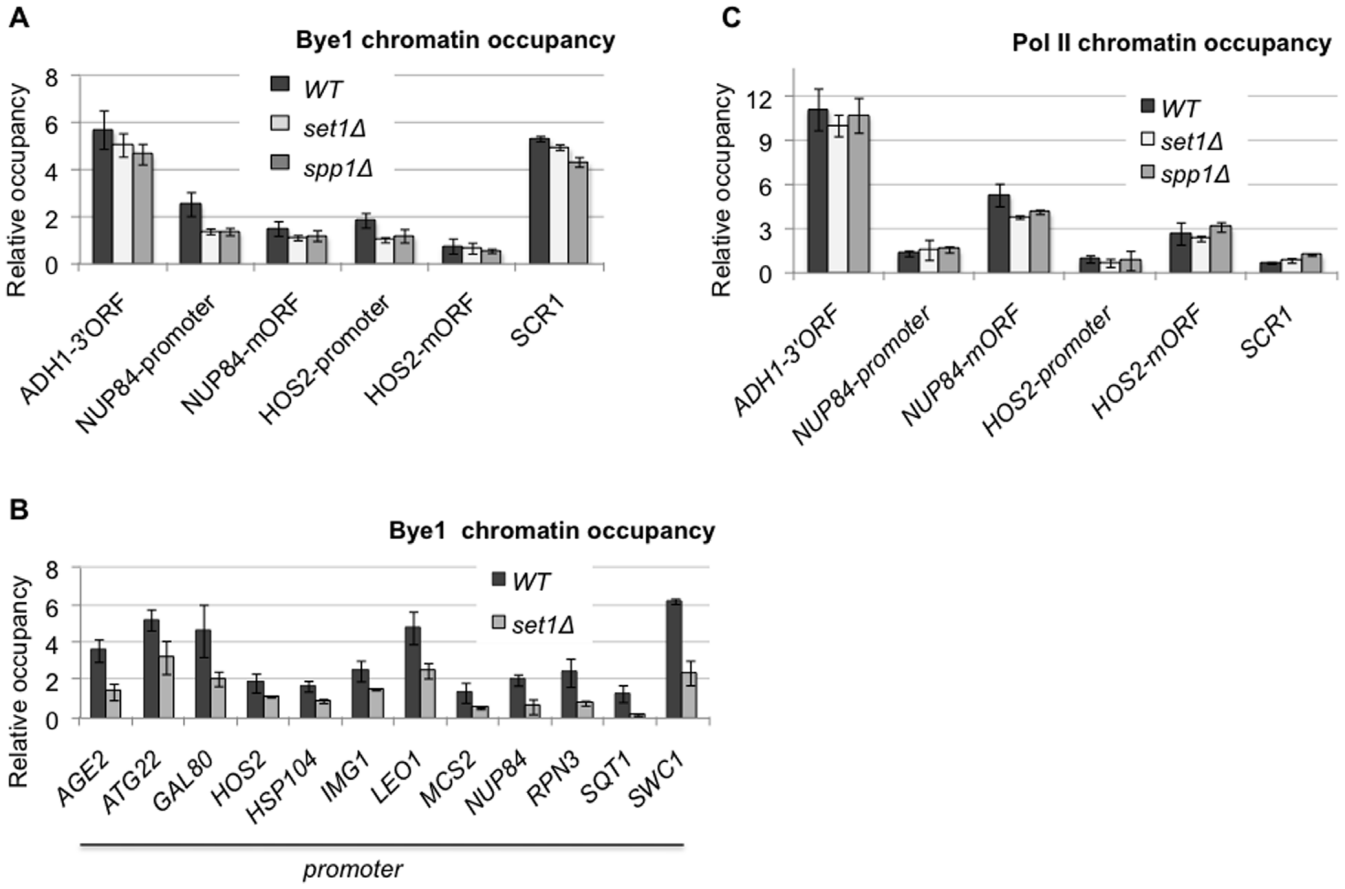
Bye1 association with promoters of class II genes requires H3K4me3: (A, B) Quantification of Bye1-Myc enrichment in *WT* and strains deleted for *SET1* or *SPP1* genes relative to the intergenic region by ChIP; (C) Quantification of Rpb1 enrichment in *WT*, *set1Δ* and *spp1Δ* strains relative to the intergenic region by ChIP. 3′ORF and mORF stand for the 3′end and middle coding regions.

Bye1 has been shown to interact with H3K4me3 *via* its PHD finger *in vitro* and, globally, to co-localize with the H3K4me3 modified +1 nucleosome downstream the TSS [17], [20]. To assess a role of H3K4 methylation in Bye1 recruitment to promoter regions *in vivo*, we performed ChIP of Bye1-Myc in strains lacking either Set1 or Spp1 subunits of the Set1C complex. Set1 is the catalytic subunit of the complex responsible for all types of H3K4 methylation (mono-, di- or tri-) while Spp1 is required only for tri-methylation of H3K4 [21]. We found that specific depletion of H3K4me3 is sufficient to abolish Bye1 binding to gene promoters tested within the dataset, but doesn’t affect it in the body of class II and class III genes (Figure 4). The ChIP-chip experiment revealed 536 class II genes showing Bye1 presence at promoters. Given the low above background Bye1 enrichment at these loci and technical limitations of microarray approaches, we manually curated 10 more promoters from this group of genes by more sensitive conventional ChIP. All of them confirmed H3K4me dependent recruitment of Bye1 to promoter regions (Figure 4B). Notably, H3K4me depletion didn’t affect Pol II occupancy upon normal growth conditions (Figure 4C).

Coding regions of genes are marked by Set2-mediated methylation of histone H3 lysine 36 essential for histone exchange and deacetylation controlling the accuracy of Pol II transcription [22]. We examined the recruitment of Bye1 to promoters and coding regions of genes in a *SET2* deleted strain. This mutant had no detectable transcriptional defects in normal growth conditions and didn’t affect Bye1 chromatin occupancy (Figure S5).

To summarize, Bye1 presence at class II gene promoters depends on Set1C activity, necessitates the Spp1 subunit and, hence, specifically requires H3K4me3 *in vivo*. On the contrary, Bye1 presence within gene bodies does not depend on H3K4 methylation.

### Bye1 shows genetic interactions with TFIIS upon stress conditions

**Figure 5:**
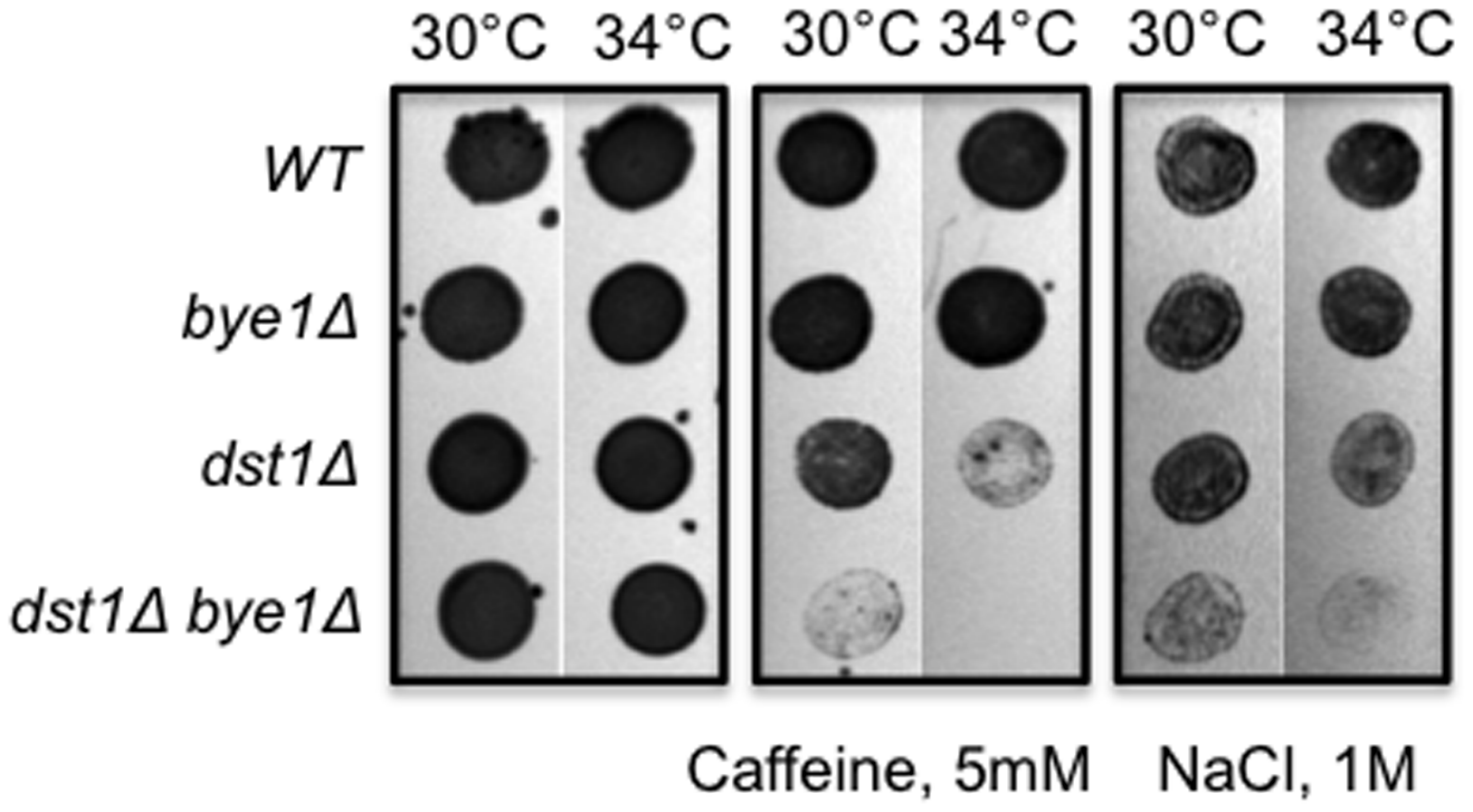
Bye1 and TFIIS exhibit a synthetic growth defect in response to high concentrations of NaCl and caffeine.

Deletion of *BYE1* alone has no discernible growth defect in multiple conditions (temperatures: 16°C, 30°C, 37°C; metabolic stress: raffinose, galactose, ethanol; osmotic stress: NaCl, caffeine; presence of nucleotide-depleting drugs: mycophenolic acid and 6-azauracil; DNA damaging treatments: UV, H_2_O_2_) (data not shown). These observations suggested that Bye1 is dispensable for viability and/or redundant with other transcription factors. We investigated the latter possibility, looking for a synthetic growth defect in a strain deleted for *BYE1* and *DST1*, the gene encoding TFIIS. A synthetic growth defect was revealed in a *bye1* and *dst1* double mutant strain in conditions of high NaCl (1M) or caffeine (5 mM) – both treatments that challenge cell wall integrity. The sickness was further enhanced at 34°C (Figure 5). The synthetic growth defect suggested that these two genes function in parallel and/or in compensatory regulatory pathways.

Caffeine is known to induce pleiotropic responses in yeast, including activation of the TOR, PKC and Ras/cAMP signalling pathways with marked changes in transcriptome composition [23]. Bye1 is present at promoters of some caffeine-induced genes (*ARO80*, *SNQ2*) even when they are transcribed at very low basal levels in the absence of the drug. We didn’t find any difference in transcriptional response to the caffeine treatment in strains deleted for *BYE1* and/or *DST1* when measuring steady-state mRNA levels for these two caffeine-induced genes (data not shown).

### Bye1 is implicated in the regulation of transcription of galactose-induced genes

**Figure 6:**
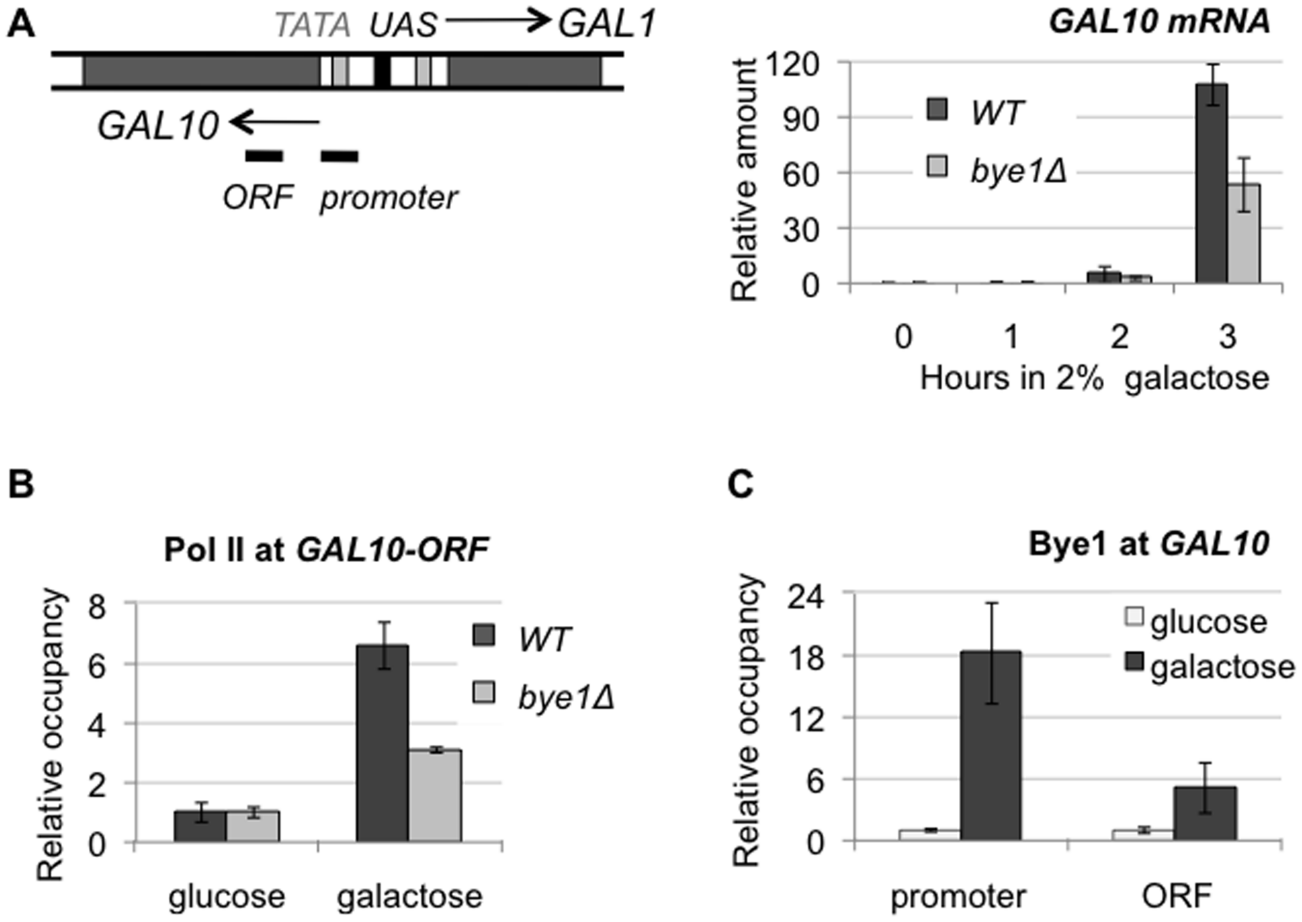
Bye1 positively regulates the *GAL10* gene transcription: (A) *GAL10* steady-state mRNA levels measured by RT-qPCR in *WT* and *bye1Δ* strains grown in a glucose- or galactose-supplemented medium; (B) Quantification of Rpb1 occupancy of *GAL10* in glucose and after 3 hours of induction in galactose media in *WT* and *bye1Δ* strains by ChIP; (C) Quantification of Bye1-Myc occupancy of promoter and coding regions of *GAL10* relative to *RPO21-*ORF under repressed (in glucose) and activated (in galactose) conditions by ChIP.

We found that the deletion of *BYE1* did not change Pol II or TBP occupancy and transcription of all genes tested in this study upon normal growth conditions (Figure S6), but does affect *GAL* genes galactose-mediated induction. *GAL1 and GAL10* genes are divergently transcribed genes sharing the same upstream activating sequence (UAS). The expression of *GAL* genes is controlled by carbon sources in the growth media, being repressed by glucose and transcriptionally activated by galactose. We demonstrated that *BYE1* deletion resulted in slower and less efficient induction of transcription as monitored by measurements of steady-state mRNA levels by RT-qPCR and the Pol II occupancy of *GAL10* by ChIP (Figure 6A and 6B). We further assessed by ChIP if Bye1 was recruited to the *GAL10* promoter and gene body (ORF) in repressed and activated conditions (Figure 6C). As expected, upon repression (in glucose) there was no detectable presence of Bye1 over the gene. On the contrary, activation of transcription (in galactose) resulted in Bye1 recruitment to both the promoter and coding regions of *GAL10*. These findings strongly argue for a positive role of Bye1 in activated transcription of *GAL* genes.

## Discussion

In eukaryotes, the TFIIS transcription factor plays a key role in assisting Pol II and, possibly, Pol III machineries to pass impediments such as nucleosomes, chromatin-binding proteins or DNA lesions in transcribed loci. Deletion of TFIIS increases Pol II pausing, compromising a release of arrested Pol II, and leads to lethality upon several stresses. In yeast, Bye1 has a Pol II-binding domain homologous to that of TFIIS, suggesting competitive or complementary functions in the transcriptional process. So far, despite the high conservation of Bye1 in the eukaryotic kingdom, its role in transcription has remained elusive. To gain a better understanding of the Bye1 function, we have undertaken a study of Bye1 chromatin occupancy genome-wide. Our results revealed a multivalent distribution of Bye1, firstly, within coding regions and a subset of promoter regions of Pol II-transcribed genes and, unexpectedly, within Pol III-transcribed genes. We focused our investigation on class II genes. In this case, Bye1 recruitment to chromatin requires active transcription. The *rpb1-1* mutation in the catalytic subunit of Pol II abolishes transcription at 37°C and leads to a release of Bye1 from chromatin. Moreover, by means of co-immunoprecipitation and yeast two-hybrid assays, we demonstrated that Bye1 interacts with Pol II *in vivo*. This result reinforces structural data showing the direct binding of the Bye1 TFIIS-like domain to the jaw domain of Pol II *in vitro* [17]. The genome-wide distribution analysis demonstrated the concurrent presence of Bye1 and TFIIS within actively transcribed gene bodies that is increased together with Pol II occupancy and gene expression levels. Hence, TFIIS and Bye1 could compete for the binding to the same module of Pol II. Interestingly, double deletion of *BYE1* and *DST1* results in growth defects upon certain stress conditions suggesting that these proteins act in parallel and/or compensatory pathways.

Taken together, our results strongly argue in favor of a role of Bye1 in transcription elongation where it could bind to the paused Pol II machinery and displace TFIIS, hence ensuring Pol II reactivation and a resumption of the transcriptional process. Such successive Bye1-TFIIS association could be important upon stress when a rapid transcriptional response is required.

In addition to its role in transcription elongation, Bye1 has a function when recruited to promoters of a subset of class II genes. We noticed that, globally, these genes are transcribed at a quite low level in normal growth conditions, but can be activated upon stress (*e.g. SQT1, HSP104, NUP84*). This feature led us to propose that the loading of Bye1 to chromatin at the initiation or early elongation steps is important for efficient transcription activation. Indeed, in the absence of Bye1, *GAL* genes are induced more slowly and less efficiently. Previous studies have shown that the *GAL* locus is transcribed under repressed conditions into long non-coding RNAs [24], [25]. This cryptic transcriptional activity results in H3K4 di-methylation within the *GAL10* gene body that further interferes with *GAL* activation. We assume that Bye1 enters the H3K4me-dependent regulatory circuit later on after the glucose to galactose switch during the first rounds of *GAL* gene transcription, allowing H3K4 tri-methylation of the TSS-proximal nucleosome. Recently, analysis of the native Pol II associated transcripts revealed that *in vivo* Pol II tends to stall when it encounters nucleosomes or other barriers [1]. It would be worth examining whether Bye1 plays any role in abortive transcription in different stress conditions to understand its role in post-initiation control and elongation.

As mentioned above, Bye1 and TFIIS are also present on genes transcribed by the Pol III machinery. One of the two Pol III subunits forming its catalytic center, Rpc160, is homologous to Rpb1 and has a similar jaw domain. So far, no structural study has addressed an interaction of TFIIS with Rpc160 and its role in class III gene transcription remains unclear. In view of our work and published data, it seems plausible that TFIIS and Bye1 compete for binding to Pol III within its jaw. However, additional experiments are required to prove a possible role of Bye1 in regulation of transcription of class III genes.

Importantly, Bye1 has two human homologues possessing the same domain organization: PHF3 and the Dido gene encoding three proteins Dido1, 2 and 3, described as putative transcription factors and abnormally expressed in glioblastoma and myeloproliferative disorders [26], [27]. Recently, Dido3 has been shown to regulate the expression of stemness genes in embryonic stem cells through its PHD finger [28]. Interestingly, phosphorylation of threonines 3 and 6 of histone H3, modifications closely linked to chromatin condensation during mitosis, abolishes the binding of human Dido3 and yeast Bye1 to H3K4me3. In human cells, they result in the translocation of Dido3 from chromatin to the mitotic spindle, where the protein seems to control microtubule organization [29]. The PHD finger of PHF3 contains mutations that render this domain incompatible with H3K4me3 binding. Nonetheless, this protein is thought to contribute to glioblastoma development since its expression is significantly diminished or lost in this type of brain tumor. The high conservation of the TFIIS-like domain between Bye1 homologues in human and yeast points to a highly conserved function in transcriptional control based on association with Pol II and/or Pol III machineries. The PHD finger and the SPOC domain could be crucial in specific targeting of Bye1 homologues to particular genomic loci to ensure their role in stress response, cell cycle regulation and development.

## Materials and Methods

*Yeast strains, oligonucleotides and plasmids* are all described in Table S6. Gene deletions and epitope-tags were introduced by transformation of Longtine-templated PCR fragments generated with specific primers (sequences can be obtained upon request) [30]. Plasmids for Y2H were prepared by the GATEWAY technique [31].

*Growth media* were prepared with standard methods using YP and CSM media (Gibco) supplemented as indicated and containing 2% glucose or 2% galactose, NaCl (1 M, Sigma), Caffeine (5 mM, Sigma).

*RNA extraction and RT-qPCR* – Total RNA was extracted using the hot phenol extraction procedure. Reverse transcription was performed with 0.1–1 µg of total RNA and BioRad iScript kit. cDNA quantifications are the mean of at least 3 independent experiments. qPCR results were normalized using *RPO21* as an internal control. Primers within the coding sequence of genes are listed in Table S6.

*Chromatin immunoprecipitation (ChIP)* – was performed as described previously [32]. 200–500 µg of sonicated chromatin was immunoprecipitated during two hours in presence of IgG magnetic beads (Dynabead) coupled with specific antibodies against H3K4me3 (Diagenode), the C-terminal domain of Rpb1 (Covance, 8WG16), the HA (Roche, 12CA5) and the Myc epitopes (Roche, 9E10). Immunoprecipitated DNA quantification is expressed as % of input DNA or relatively to a specific genomic region (*RPO21-ORF* or Intergenic Region). Primer sequences are listed in Table S6. Error bars correspond to standard deviations of at least three independent experiments.

*Co-immunoprecipitation* – was performed as for ChIP. After IP and extensive wash beads were boiled in 2xSDS-PAGE loading buffer and loaded to 10% SDS-PAGE. Western blot was performed using the same antibodies as for IP. IP without antibodies was used as a Mock control.

*Microarray hybridization* – was done with a use of Agilent Yeast Whole Genome ChIP-chip Microarray 4×44 K 014810 G4493A. Three biological replicates of Bye1-Myc ChIP-chip and four biological replicates of Pol II and TFIIS ChIP-chip have been used for all quantifications. The computational data analysis was performed in R using limma package from the Bioconductor project [33] as described [12]. Raw data have been deposited to Array Express under accession number E-MTAB-2161.

*Yeast two-hybrid assay –* was performed in the Y190 strain transformed with pVV212 and pVV213 empty or recombinant vectors listed in Table S6, and the activation of the *lacZ* or *HIS3* reporters was tested using X-Gal overlay plate assay or 3AT plate assay as described [34], [35].

## Supporting Information

Figure S1 **Distribution of Pol II, TFIIS and Bye1 along class II genes: (A)** ***ADH1 and PYK1*** **, (B)** ***NUP84*** **and** ***HOS2*** **as assessed by ChIP-chip assay.**
(TIF)

Figure S2 **Distribution of Pol II, TFIIS and Bye1 along class III genes:** ***tL(UAA)B1*** **and** ***SCR1*** **as assessed by ChIP-chip assay.**
(TIF)

Figure S3 **Bye1-Myc protein level doesn’t change after abolishment of Pol II transcription following 30 min heat shock of the** ***rpb1-1*** **mutant strain measured by Western blot.**
(TIF)

Figure S4 **Bye1 recruitment to class II genes depends on gene expression levels: (A) Genome wide dot-plot analysis of Bye1 fold enrichment and (B–D) scatter-plot analysis of Bye1-Myc and Pol II fold enrichment of coding regions of class II genes expressed at low (<1 copy/cell), middle (from 1 to 10 copies/cell) and high (more than 10 copies/cell) levels as assessed by ChIP-chip in the Bye1-Myc HA-TFIIS strain with Pearson (** ***r*** **) and Spearman (** ***ρ*** **) correlation coefficients.**
(TIF)

Figure S5 **Deletion of** ***SET2*** **has no significant effect on Pol II and Bye1 recruitment to chromatin: Quantification of Pol II (A) and Bye1-Myc (B) enrichment of different genomic loci in** ***WT*** **,** ***set1Δ*** **and** ***set2Δ*** **strains relative to the intergenic region by ChIP.** 3′ORF and mORF stand for the 3′end and middle coding regions.
(TIF)

Figure S6 **Deletion of** ***BYE1*** **doesn’t affect Pol II and TBP recruitment to class II genes in normal growth conditions: Quantification of (A) Pol II and (B) HA-TBP chromatin occupancy in** ***WT*** **and** ***bye1Δ*** **strains by ChIP. 3′ORF and mORF stand for the 3′end and middle coding regions.**
(TIF)

Table S1 **Bye1 occupancy of the yeast genome.**
(XLS)

Table S2 **Pol II occupancy of the yeast genome.**
(XLS)

Table S3 **TFIIS occupancy of the yeast genome.**
(XLS)

Table S4 **Genes showing a significant Bye1 occupancy of promoter and 5′-coding regions (fold enrichment ≥2 and the p value ≤0.05).**
(XLS)

Table S5 **GO term annotations for biological processes of genes showing Bye1 presence at promoters**
(XLS)

Table S6 **List of primers, plasmids and strains used in this study.**
(XLSX)

Table S7 **List of class III genes examined in this study.**
(XLSX)
