# Supplementary figures and images for: PHD and TFIIS-Like Domains of the Bye1 Transcription Factor Determine Its Multivalent Genomic Distribution
Source: PLoS One. 2014 Jul 16;9(7):e102464. doi: 10.1371/journal.pone.0102464 (PMC4100922; doi:10.1371/journal.pone.0102464)

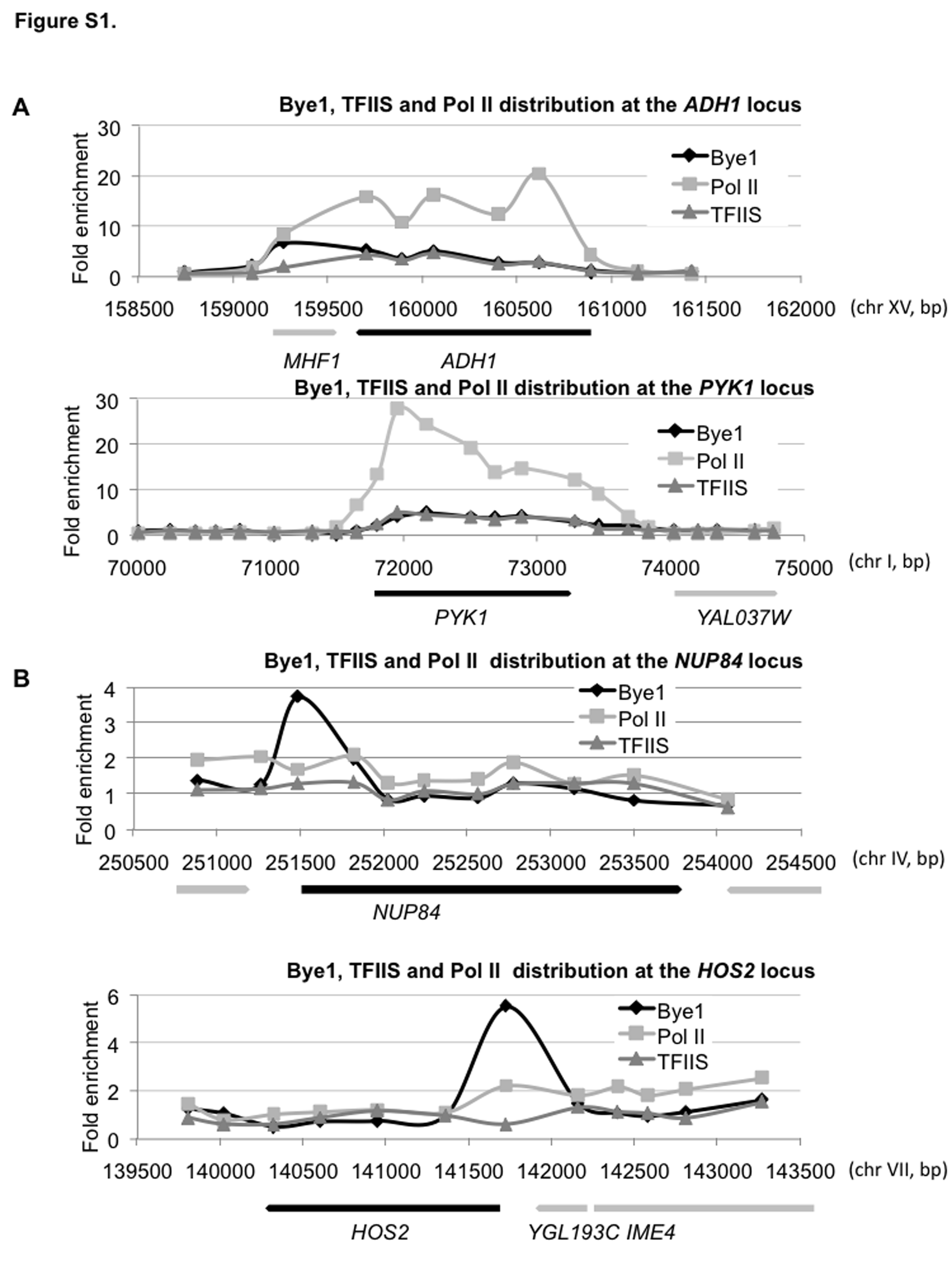

Supplement: Figure S1 — Distribution of Pol II, TFIIS and Bye1 along class II genes: (A) ADH1 and PYK1 , (B) NUP84 and HOS2 as assessed by ChIP-chip assay. (TIF) [file pone.0102464.s001.tif]

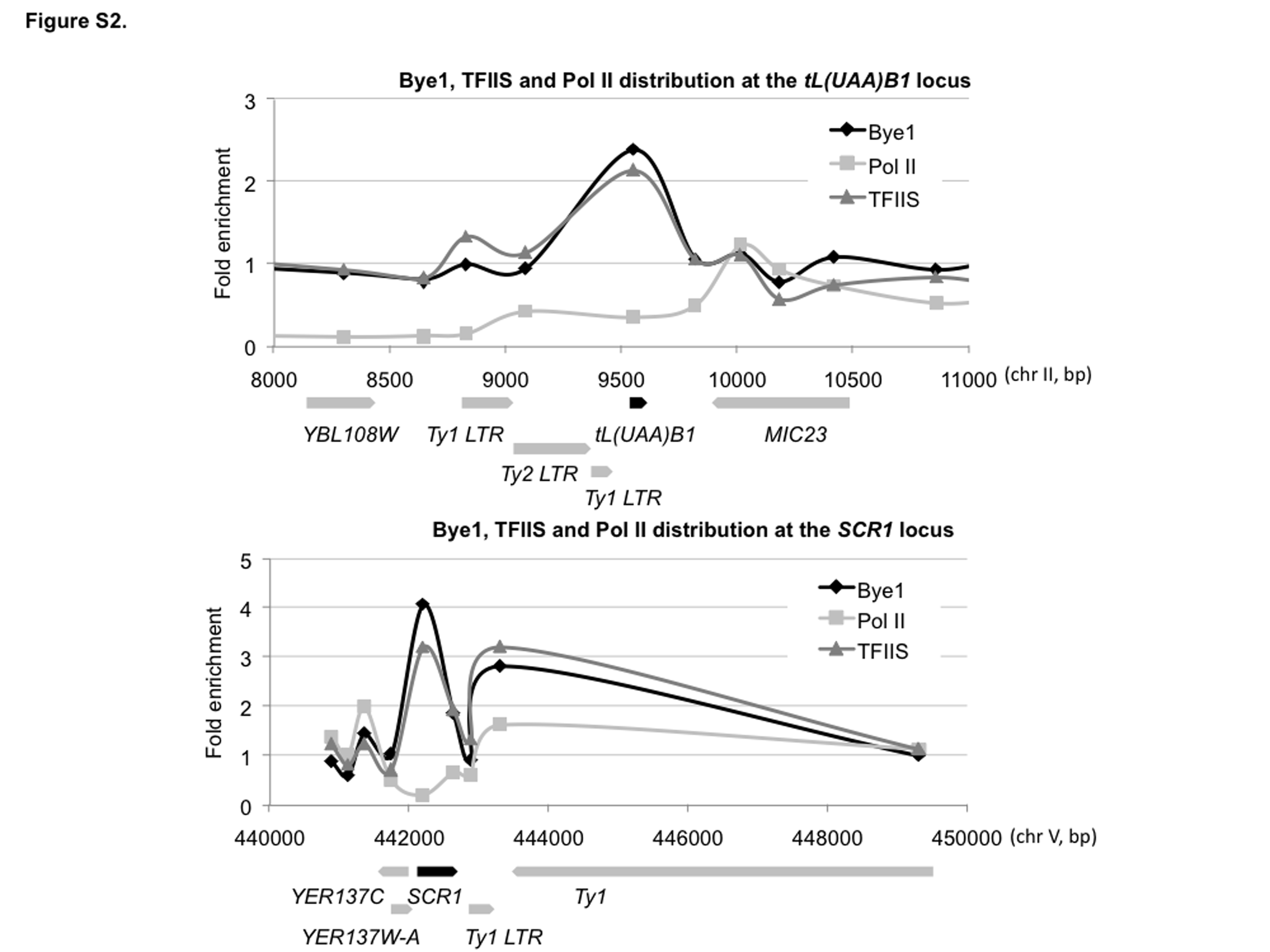

Supplement: Figure S2 — Distribution of Pol II, TFIIS and Bye1 along class III genes: tL(UAA)B1 and SCR1 as assessed by ChIP-chip assay. (TIF) [file pone.0102464.s002.tif]

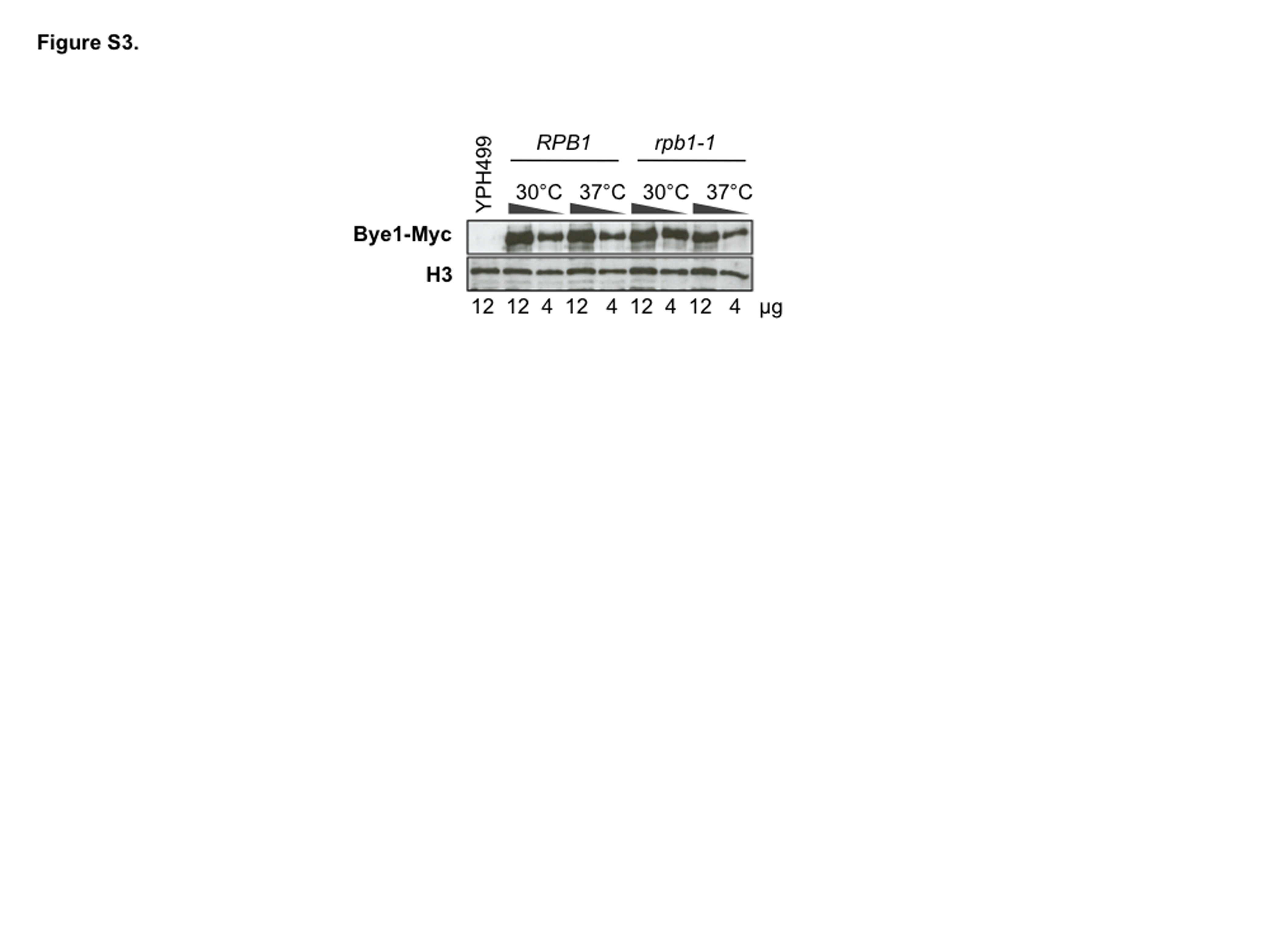

Supplement: Figure S3 — Bye1-Myc protein level doesn’t change after abolishment of Pol II transcription following 30 min heat shock of the rpb1-1 mutant strain measured by Western blot. (TIF) [file pone.0102464.s003.tif]

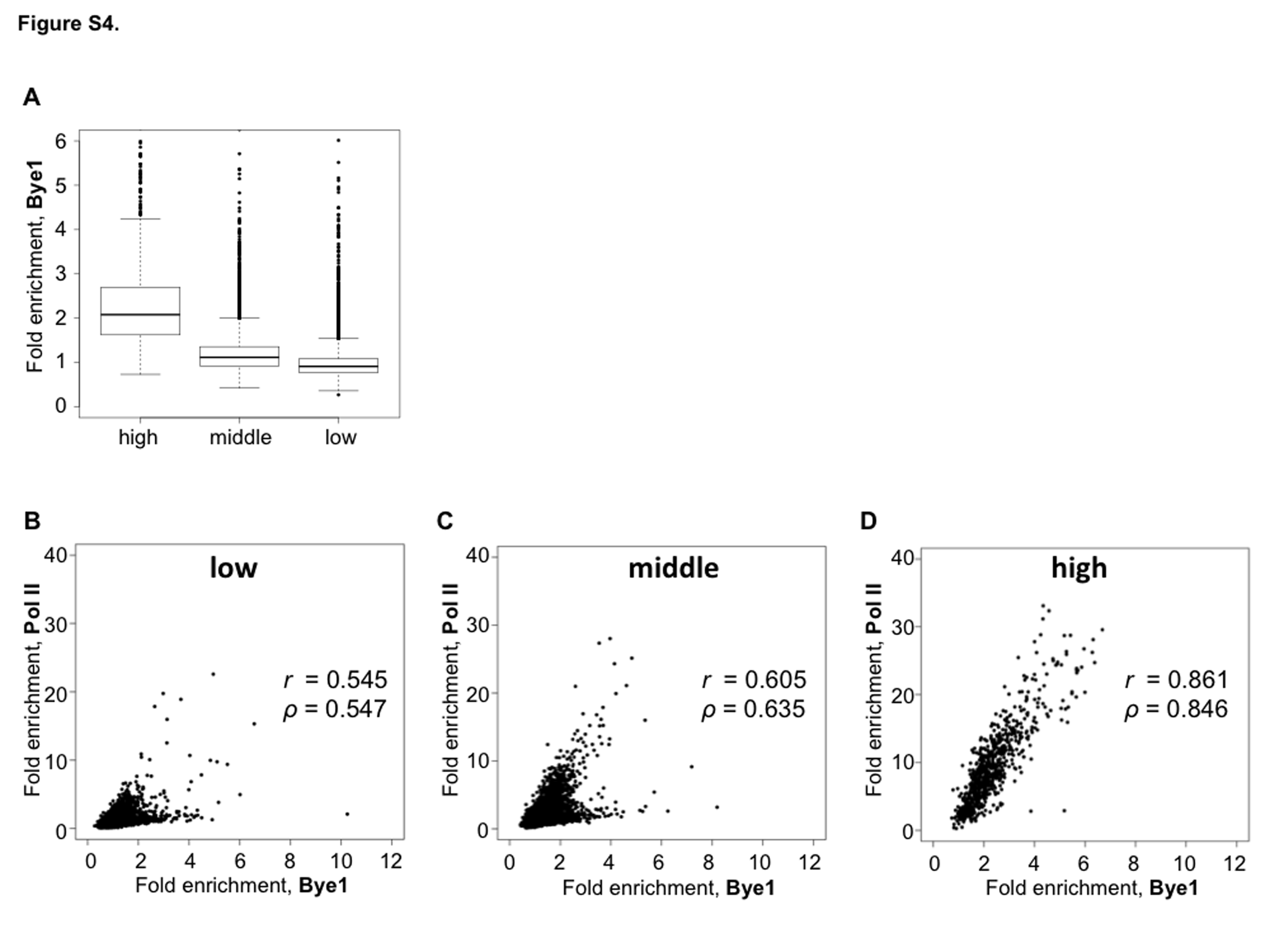

Supplement: Figure S4 — Bye1 recruitment to class II genes depends on gene expression levels: (A) Genome wide dot-plot analysis of Bye1 fold enrichment and (B–D) scatter-plot analysis of Bye1-Myc and Pol II fold enrichment of coding regions of class II genes expressed at low (<1 copy/cell), middle (from 1 to 10 copies/cell) and high (more than 10 copies/cell) levels as assessed by ChIP-chip in the Bye1-Myc HA-TFIIS strain with Pearson ( r ) and Spearman ( ρ ) correlation coefficients. (TIF) [file pone.0102464.s004.tif]

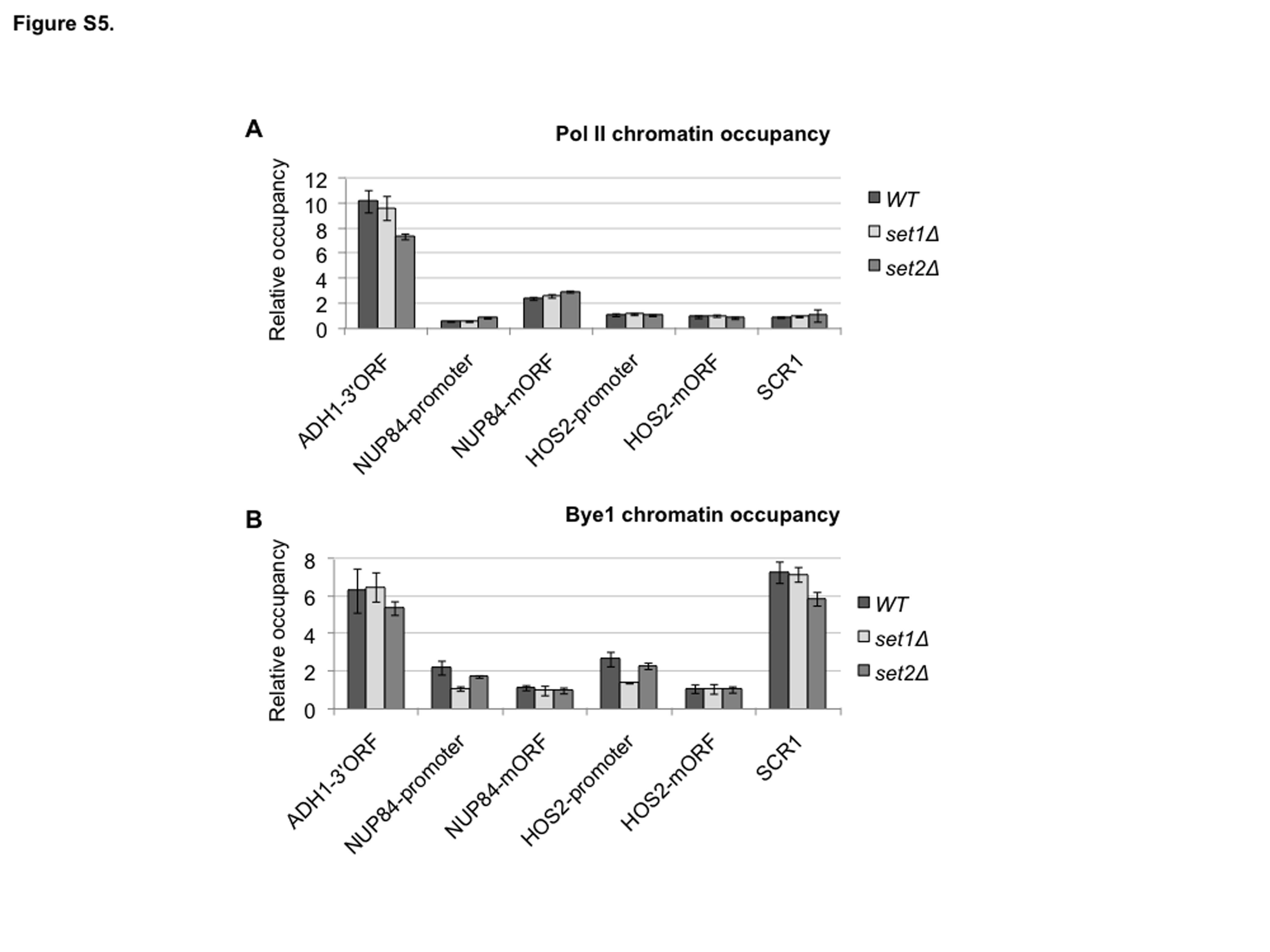

Supplement: Figure S5 — Deletion of SET2 has no significant effect on Pol II and Bye1 recruitment to chromatin: Quantification of Pol II (A) and Bye1-Myc (B) enrichment of different genomic loci in WT , set1Δ and set2Δ strains relative to the intergenic region by ChIP. 3′ORF and mORF stand for the 3′end and middle coding regions. (TIF) [file pone.0102464.s005.tif]

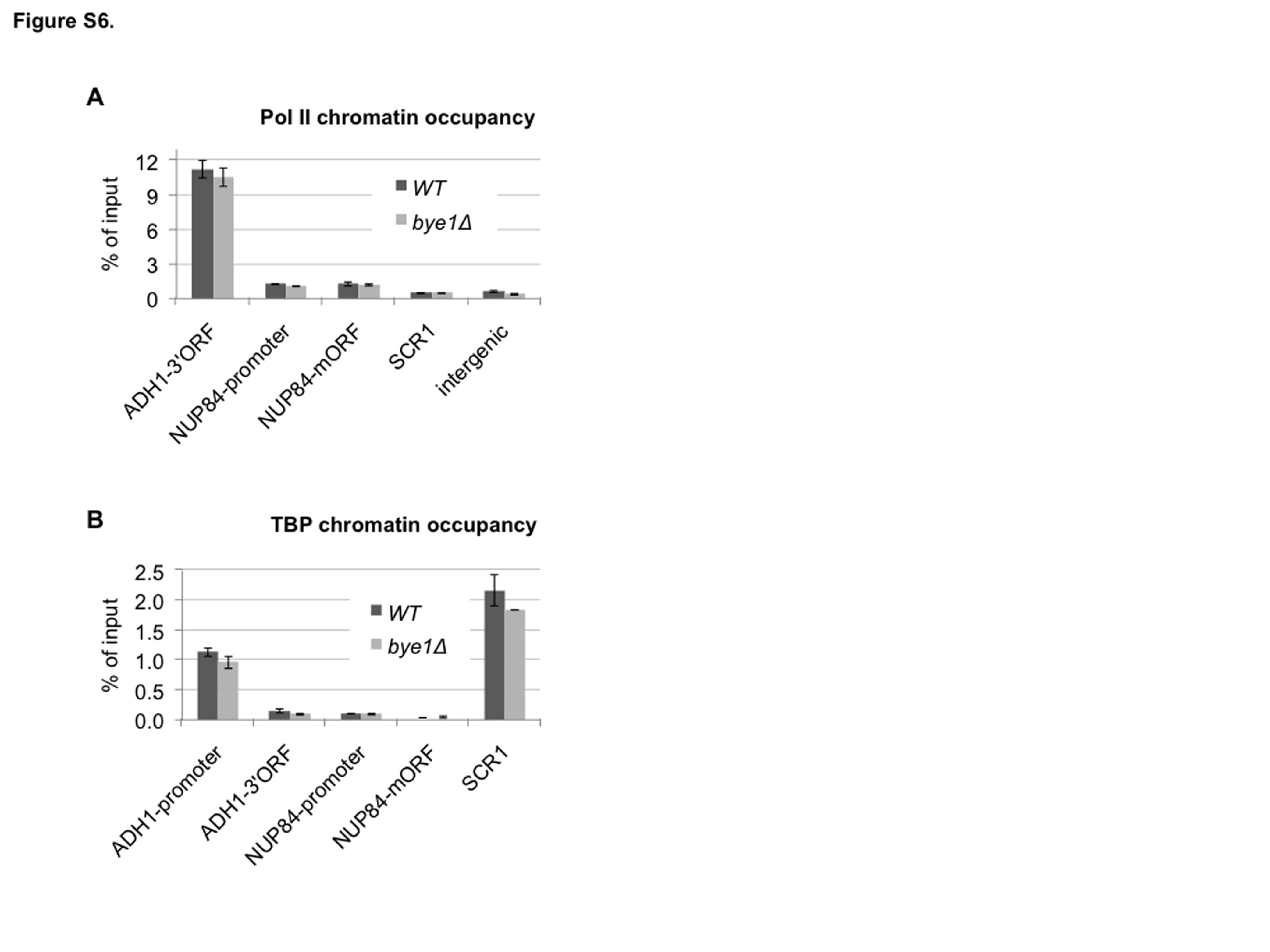

Supplement: Figure S6 — Deletion of BYE1 doesn’t affect Pol II and TBP recruitment to class II genes in normal growth conditions: Quantification of (A) Pol II and (B) HA-TBP chromatin occupancy in WT and bye1Δ strains by ChIP. 3′ORF and mORF stand for the 3′end and middle coding regions. (TIF) [file pone.0102464.s006.tif]
